# High-aspect-ratio nanostructured hydroxyapatite: towards new functionalities for a classical material

**DOI:** 10.1039/d3sc05344j

**Published:** 2023-12-01

**Authors:** Anna Diez-Escudero, Montserrat Espanol, Maria-Pau Ginebra

**Affiliations:** a Biomaterials, Biomechanics and Tissue Engineering Group, Department of Materials Science and Engineering, Universitat Politècnica de Catalunya (UPC) Av. Eduard Maristany 16 08019 Barcelona Spain maria.pau.ginebra@upc.edu; b Barcelona Research Center in Multiscale Science and Engineering, Universitat Politècnica de Catalunya (UPC) Av. Eduard Maristany 16 08019 Barcelona Spain; c Institute for Bioengineering of Catalonia (IBEC), The Barcelona Institute of Science and Technology Baldiri Reixac 10-12 08028 Barcelona Spain

## Abstract

Hydroxyapatite-based materials have been widely used in countless applications, such as bone regeneration, catalysis, air and water purification or protein separation. Recently, much interest has been given to controlling the aspect ratio of hydroxyapatite crystals from bulk samples. The ability to exert control over the aspect ratio may revolutionize the applications of these materials towards new functional materials. Controlling the shape, size and orientation of HA crystals allows obtaining high aspect ratio structures, improving several key properties of HA materials such as molecule adsorption, ion exchange, catalytic reactions, and even overcoming the well-known brittleness of ceramic materials. Regulating the morphogenesis of HA crystals to form elongated oriented fibres has led to flexible inorganic synthetic sponges, aerogels, membranes, papers, among others, with applications in sustainability, energy and catalysis, and especially in the biomedical field.

## Abstract 2

Hydroxyapatite-based materials have been widely used in countless applications, such as bone regeneration, catalysis, air and water purification or protein separation.

## Graphical abstract

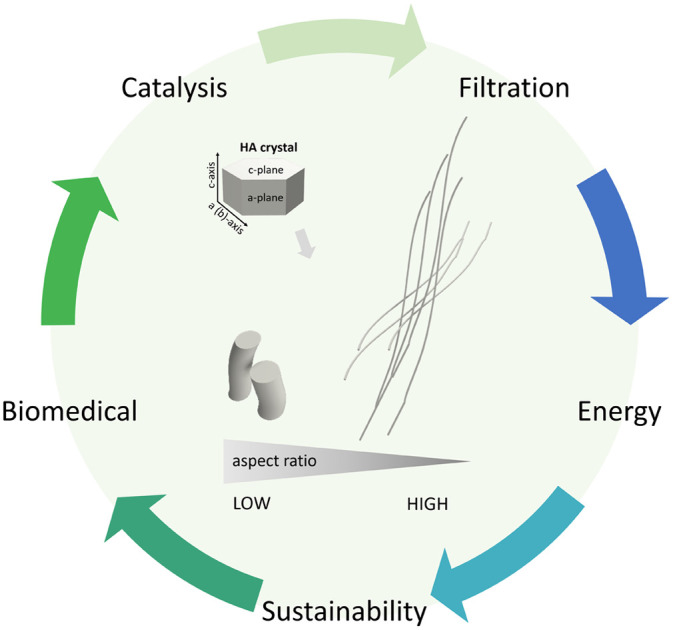

## 1. Introduction

Calcium phosphates are a family of materials, found in nature in the form of minerals and biominerals, which can also be obtained by synthetic processes, and which have found a wide range of technological applications. They are usually classified according to the acid from which they are derived as orthophosphates (salts derived from orthophosphoric acid), pyrophosphates (derived from pyrophosphoric acid) and metaphosphates or condensed polyphosphates (derived from the hypothetic metaphosphoric acid).

Calcium orthophosphates are obtained from the neutralization of the different acidities of the orthophosphoric acid (H_3_PO_4_). The progressive replacement of acidic protons by calcium ions results in different calcium salts, with Ca/P ratios ranging between 1 and 2. Among them, apatites are the most frequently encountered crystalline calcium phosphates. They are the mineral constituent of bones and teeth, and they are also among the most used calcium phosphate compounds. They are biocompatible materials, with a range of applications in the biomedical field such as bone tissue engineering and drug delivery. Moreover, apatites have been extensively studied for their catalytic and environmental remediation properties, by taking advantage of some very relevant properties such as their high adsorption capacities, the fact that they contain both acid and basic sites in their structure, their ion exchange capacity and their good thermal and chemical stability.^1^ In the field of catalysis, apatite-based materials have been used for various reactions, including condensation, oxidation and photocatalytic reactions amongst others.^2,3^ In the field of environmental remediation, apatites have been used for the removal of heavy metals, organic pollutants, and dyes from contaminated water and soil.^4^

The chemical composition of calcium phosphate apatites is given by the formula Ca_10_(PO_4_)_6_X_2_, where X can be a F^−^ ion (fluoroapatite), OH^−^ ion (hydroxyapatite) or Cl^−^ ion (chloroapatite). In this article we will focus on hydroxyapatite (HA), whose stoichiometric formula is Ca_10_(PO_4_)_6_(OH)_2_ [Ca/P = 10/6 = 1.67]. However, there are also non-stoichiometric hydroxyapatites, with Ca/P ratios lower than the stoichiometric composition, when apatites contain HPO_4_^2−^ ions in place of PO_4_^3−^ ions, which results in the formation of vacancies to preserve electroneutrality, generating a solid solution field:Ca_10−*x*_(PO_4_)_6−*x*_(HPO_4_)_*x*_(OH)_2−*x*_ with 0 ≤ *x* ≤ 1

Moreover, the apatite structure is very tolerant to ionic substitutions and can accommodate numerous cations and/or anions in its lattice.^5^ This gives great versatility to this compound and numerous studies have exploited the possibility of doping HA with various ions.^6^

However, in this article our emphasis, rather than on composition, will be on the morphology of the crystals. More specifically, we will focus on the role of the crystal aspect ratio, defined as the ratio between length and thickness of the crystals as a relevant parameter for improving the functional properties of this family of materials in their various fields of application.

This perspective article focuses on the current state of knowledge to control of the aspect ratio of HA crystals, and its effect on biomaterials, catalysis, and environmental remediation fields. We will also highlight the latest developments and future perspectives in these areas of research. The first section describes, by way of illustration, nature's use of this parameter in the design of HA which forms the mineral phase of two mineralised tissues, bone and tooth enamel. The second section discusses in general terms the implications of the aspect ratio on the physical, chemical and mechanical properties of the material. The third section covers the synthesis methods that have been developed to tune the aspect ratio of HA crystals, including biomimetic, hydrothermal and solvothermal methods. The fourth section is devoted to the applications that benefit from high aspect ratio HA, highlighting the distinct advantages that this parameter brings to the performance of the materials. Different fields of application are covered, such as catalysis, bioimaging, fabrication of flexible ceramics, pollution treatment, and special attention is given to medical applications, where the effect on ionic, molecular and cellular interactions plays a relevant role. Finally, the final section presents conclusions and future perspectives.

## 2. Form follows function. The example of hydroxyapatite in bone and dental enamel

More than a century ago, the distinguished Chicago school architect Louis Sullivan coined the principle that “form follows function”.^7^ This motto has inspired countless designers, architects and engineers. It can also be applied to the structural design of materials, tuning their geometrical features with the aim of tailoring their properties and obtaining new functionalities.

Nature represents a good example of this principle. Bone and dental enamel are mineralised tissues, where HA is the main component. Their functions and properties are very different. Whereas bone mineral must integrate into the collagen matrix and dissolve under controlled conditions to facilitate a high rate of bone remodelling and release the bioinorganic elements (Ca and P among others) essential for metabolism and homeostasis, enamel must exhibit great stability to resist abrasion and acid attack. Controlling the size and shape of apatite crystals is one of the strategies that allows adjusting to these disparate functional needs. While in bone the apatite crystals have the form of nanometric plates a few nanometers thick and 25 to 50 nm wide, in enamel, apatite crystals grow in prismatic shape, forming much larger needles that can reach several microns in length.^8,9^ In addition, the presence of an organic phase in different proportions and configurations also contributes to the diversity of properties between these two tissues. In bone, the close interaction of HA nanoplatelets with the collagen fibres, which account for a total of 30 wt%, is responsible for the excellent combination of strength and toughness. On the contrary, dental enamel, which has less than 2 wt% of organic matter, the specific configuration of the aligned apatite rods, increases the damage tolerance of enamel through various toughening mechanisms.

**Fig. 1:**
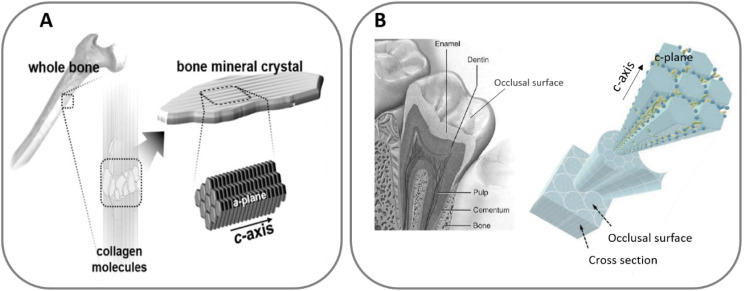
HA *c*-axis oriented in bone (A), adapted with permission from ref. 16, copyright 2020 American Chemical Society; and HA orientation in enamel (B), adapted with permission from ref. 13 and 17, copyright 2020, 2009 Elsevier.

Moreover, although in both tissues HA crystals grow in the *c*-axis direction, the exposed faces are different in the two cases. While in bone the platelets are oriented with the *c*-axis parallel to the collagen fibres and the (*a*, *b*)-planes are interfacing the collagen, in enamel the crystals are oriented perpendicularly to the surface, which results in the *c*-planes exposed to the enamel occlusal surface (Fig. 1). These variations in configuration lead to changes in the physical properties of the surface, such as wettability and surface charge, which in turn affect the interaction with the ions and proteins present in the physiological fluids.^10–15^

Overall, the impact of the different configuration of the biological apatite crystals in bone and teeth illustrates the relevance of crystal morphology as a design parameter capable of tailoring the properties and final performance of hard tissues, which can be also extended to the more general context of materials engineering.

## 3. Relevance of the aspect ratio at different length scales. Porosity, surface properties and mechanical properties

**Fig. 2:**
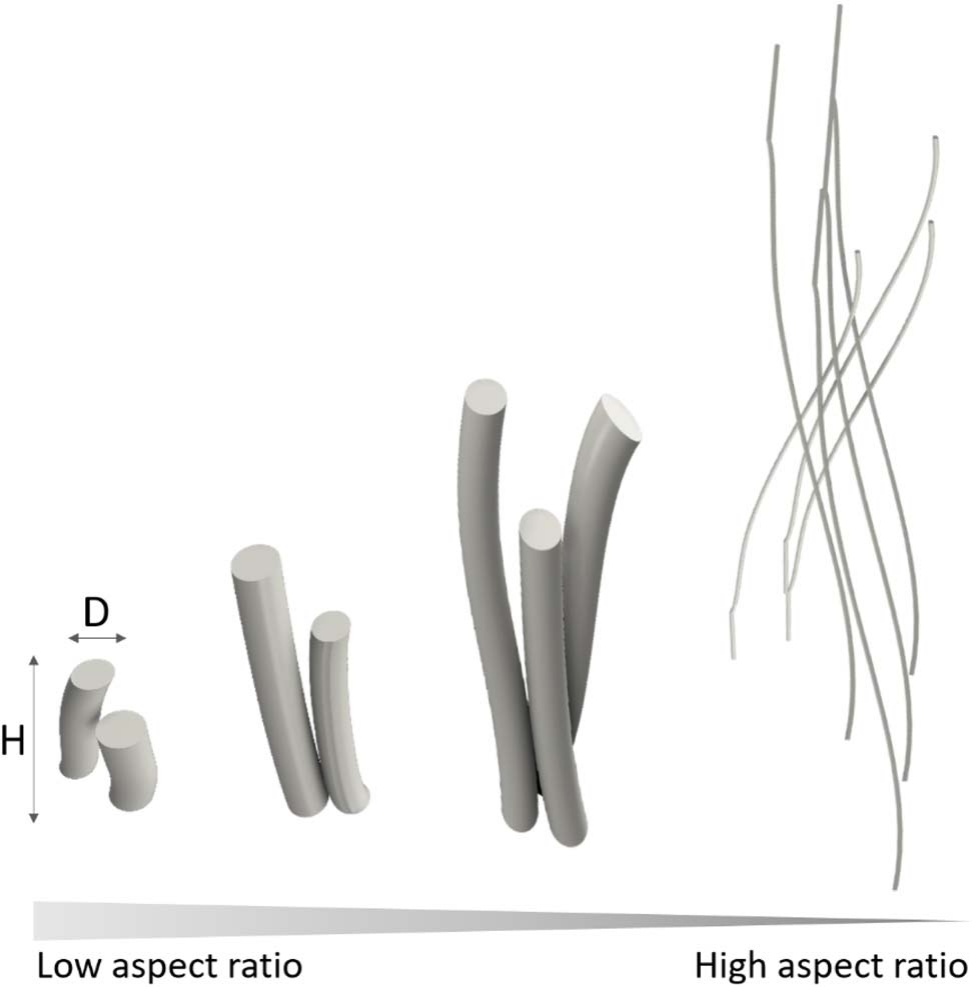
Diameter (*D*) and height (*H*) schematic defining low and high-aspect-ratio.

The morphology, or the external shape and surface features of a crystal are important characteristics that can affect the chemical, physical and mechanical properties of a material. In this article we will focus on a specific trait of crystal morphology, the aspect ratio, defined as the ratio of its length to its width (*H*/*D*, Fig. 2), with special attention to high-aspect-ratio nanostructures. There is no explicit definition in the literature of what is considered a high-aspect-ratio. In fact, the threshold for defining a high aspect ratio nanostructure is highly dependent on the application; for instance, aspect ratios of 5 : 1 or 10 : 1 are considered high in bone tissue engineering, drug delivery applications or diffusion and adsorption applications. In contrast, the thresholds for achieving structural advantages such as highly flexible ceramics are much higher, requiring aspect ratios of 100 : 1 or larger, as highlighted by Li,^18^ who showed that HA nanowires with aspect ratio of 65 : 1 were barely flexible compared to 100 : 1 aspect ratio nanowires. In this article we will use the less restrictive threshold of 10 : 1 (ref. 19) to cover more fields of application.

**Fig. 3:**
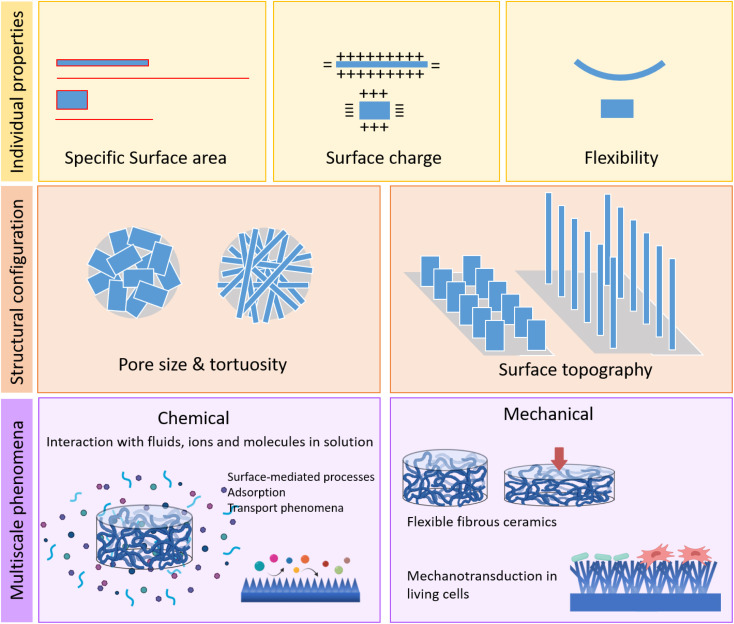
Relevance of the aspect ratio on the properties of the individual crystals and in 3D nanostructured polycrystalline materials.

The impact of the aspect ratio on the properties of individual crystals and in polycrystalline materials at different length scales, as well as their influence in different multiscale phenomena is summarised in Fig. 3.

The size and shape of the crystals are linked to the specific surface area of the material, and therefore to its reactivity. The size, especially the nanometer size of the crystallites, provides very high ratios of surface area to volume, which are magnified when the crystals have high-aspect-ratio, imparting high dissolution rates and surface activities. In addition, the exposure of different crystal faces, which depends on the crystal growth habit, determines other properties such as surface charge and surface energy of the crystal. And from a mechanical perspective, the synthesis of crystals with high-aspect-ratio results in structures with high flexibility, unusual for inorganic materials.

The shape of the crystals, and their aspect ratio, as well as their spatial organization determine also the textural properties of polycrystalline materials, such as pore size, geometry and tortuosity.^20^ At surface level, it allows obtaining nanostructures with controlled topographies, with the capacity to interact in specific ways with cells or bacteria, through various forms of mechanotransduction phenomena.

The set of properties described above have very relevant effects in diffusion-mediated and surface-mediated processes, such as adsorption phenomena of fluids such as gases and liquids, as well as for the interaction with ions or molecules in solution. These phenomena often mediate chemical processes that are very relevant in diverse scenarios, from catalysis of chemical reactions to the interactions of biomaterials with the body fluids that mediate the cell and tissue response. Finally, the possibility of tuning the aspect ratio of the crystals also opens new possibilities in the design of materials with unique mechanical properties, such as flexible ceramics.

## 4. Synthesis of high-aspect-ratio nanostructured hydroxyapatite

### 4.1. Crystal size and shape control in hydroxyapatite synthesis

#### 4.1.1. General aspects controlling crystal nucleation and growth

In recent decades much research has been devoted to the synthesis methods and applications of HA nanoparticles, including the control of their shape and size, with the consequent implications for their applications, which has been reported in some excellent reviews.^21,22^ This section will provide a brief explanation on how crystal shape is controlled in nanoparticles to later understand the development of nanostructured HA materials focusing on the effects that the control of the aspect ratio of the crystals has on the functionality of the materials.

**Fig. 4:**
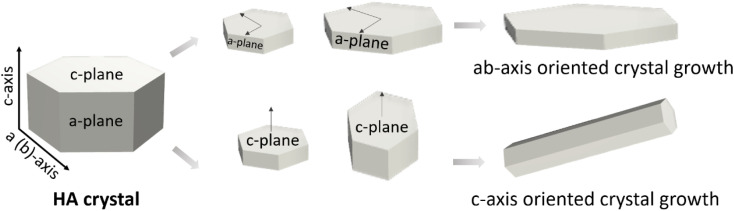
Typical hexagonal HA crystal structure with the possible orientation growth on *a*, *b* or *c* planes.

HA usually crystallises in a hexagonal structure and belongs to the *P*6_3_/*m* space group (Fig. 4), with *ab*-planes rich in calcium ions and therefore positively charged, and *c*-planes rich in phosphate and hydroxyl ions and therefore negatively charged.

It is important to note, however, that the crystal growth direction is not univocally linked to the crystal morphology. That is, growing in the *c*-direction does not necessarily imply having a prismatic needle-like morphology. This is the case, for example, of the apatite crystals in bone, which despite having a *c*-axis orientation, have a plate-like morphology, with preferential exposure of the *a*–*b* faces.

**Fig. 5:**
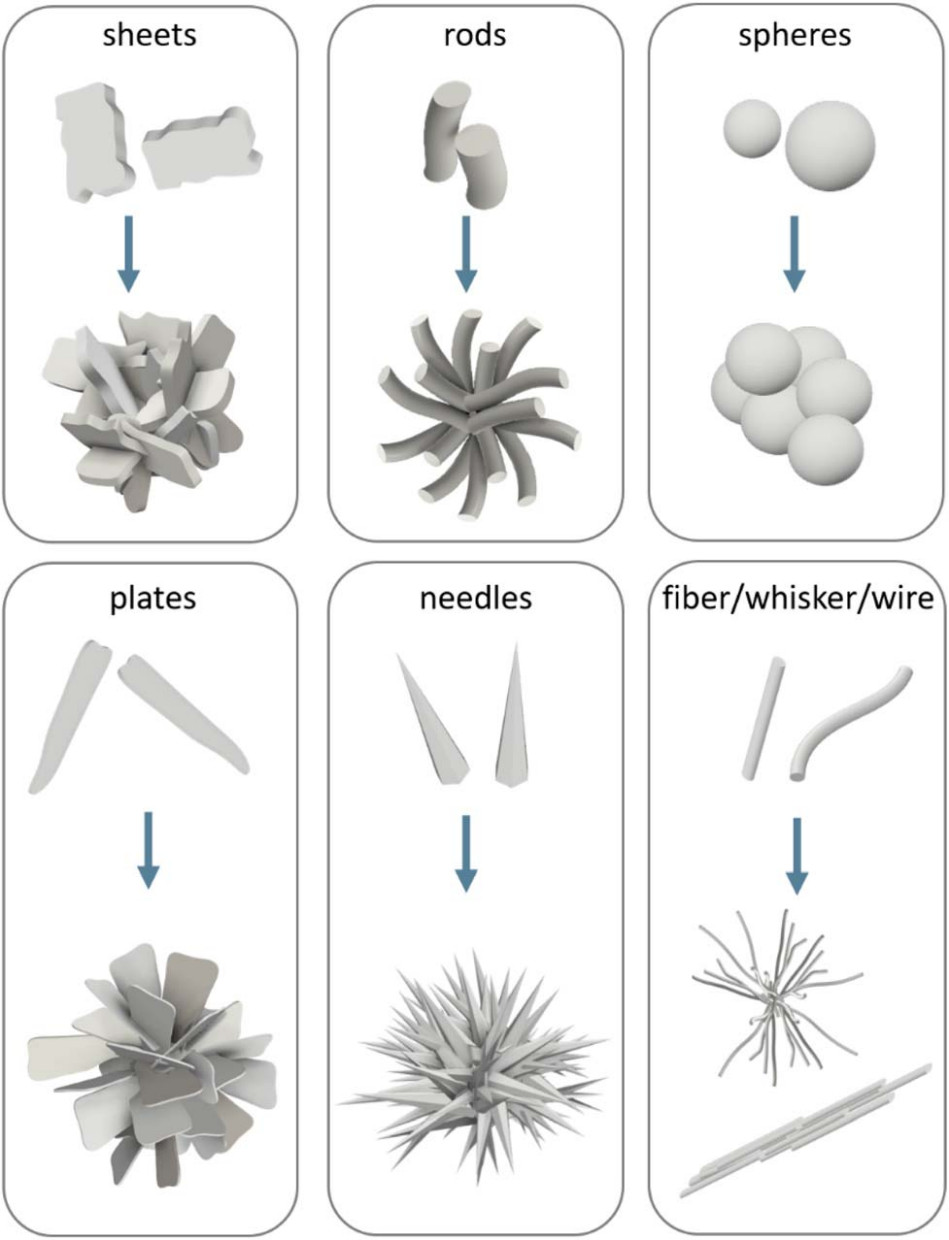
Schematic representation of the main crystal shapes obtained for HA and their possible assembly conformation.

HA has been synthesised in many shapes, including spheres, plates, needles, rods, sheets, whiskers, fibres or wires (Fig. 5). To understand the morphological control of crystals, it is necessary to understand the mechanisms of crystal formation. One way to visualise crystallisation is by looking at the “energy landscape”.

Crystallisation can be seen as the phase transition of matter from a state of high free energy in a solvated state to one of low free energy in the crystal lattice. The thermodynamic driving force for the formation of the solid phase is the supersaturation of the solution. This term refers to the excess of dissolved solute with respect to the solubility curve of the solid phase. Once supersaturation is achieved, then crystallisation can take place through the formation of a stable nuclei and its subsequent growth. The choice of the starting supersaturation in crystallisation studies has already an impact on the design of crystals of a desired shape as shown in the linear dependence between supersaturation and crystal growth.^23^

According to the classical nucleation theory (CNT), a crystal nucleus can form or redissolve based on the interplay between the total Gibbs free-energy associated with the formation of a new surface and the bulk energy gained from creation of a crystal lattice. The equation for the total Gibbs free-energy cost to form a spherical nucleus of radius *r* is:^24,25^

1 Δ*G* = −{[(4/3)π*r*^3^]/*Ω*}Δ*μ* + 4π*r*^2^*γ*

Δ*μ* measures the free energy response for transferring ions from one phase to the other (the larger this magnitude, the greater is the driving force for crystallization), *Ω* is the volume per molecule, and *γ* is the interfacial free energy.

In eqn (1) the first term, which is negative and proportional to the crystallite volume, accounts for the stable contribution of the “bulk” compared to the supersaturated fluid. The second term is a “surface area” term that accounts for the free energy cost involved in creating the solid–liquid interface. This term is positive and proportional to the surface area of the crystallite. Beyond a critical size, the surface energy is compensated by the bulk energy. Thus, if the nucleus grows beyond the critical size, the lattice energy gain exceeds the energy loss due to surface formation that drives the growth of the nucleus by ion attachment. The existence of a critical size allows, to a certain extent, controlling nucleation: critical size and interfacial energy are related, thus, the smaller the interfacial energy, the smaller the critical size and the more likely the occurrence of nucleation at a given saturation.^26^

Besides, crystallization can occur by homogeneous or heterogeneous nucleation. Homogeneous nucleation consists in the spontaneous formation of a nucleus from solution when a critical supersaturation is reached, whereas in heterogeneous nucleation we use a foreign surface to control nucleation. This later strategy, apart from being less energetically demanding, can be used to exert even greater control over nucleation because the interfacial energy between a crystal nucleus and a solid substrate is usually lower than that of the crystal in contact with the solution. Furthermore, if the atomic structure of the substrate surface matches a particular crystallographic plane of the nucleating phase, nucleation will preferentially occur on that crystal plane. This simple strategy can be applied for the nucleation of specific faces on chemically tailored surfaces.

An inherent consequence of the classical nucleation theory is that crystalline growth proceeds as a single-step pathway where ion attachment leads to the formation of a nuclei with an ordered crystalline structure which propagates as crystal grows. In such scenario the final morphology of the crystal is governed by the Wulff's rule^27^ that states that the equilibrium morphology will be dictated by the surface energy of each face. This means that faces with high surface energies will grow so fast that they will tend to disappear from the crystal, while faces with low surface energies will grow slowly and dominate the final shape. The ability to control the crystal shape by tuning the surface energy is a powerful tool to control the final morphology in a predictable way. In fact, by using face-selective additives, such as organic molecules or ions, or by changing the solvent, crystal morphogenesis can occur.^26,28^

One shortcoming of the classical nucleation theory is that it assumes that crystal growth occurs as a single-step pathway, however, there are many instances in which crystallization proceeds by sequential steps involving structural and compositional changes of intermediate phases (Ostwald's rule of stages) before reaching the thermodynamically stable final phase (*i.e.*, crystallization under kinetic control).^29^ According to Ostwald's rule, usually, the first precipitating phase will be the least stable and closest in free energy to the solution, followed by phases in order of increasing stability. The temporal stabilisation of these least stable phases (in many cases amorphous phases) opens up multiple reaction possibilities, such as dissolution–recrystallization, aggregation, solid-phase transformation and mesoscopic transformations, which allow many possibilities to control of crystal shape.^28^

The ultimate shape of crystals depends upon many factors both of energetic and kinetic nature. Despite the huge progress in the field, many questions remain unanswered in these dynamic processes. This is due in part to the limitations of existing experimental approaches and the lack of synthesis details in many of the experiments. Nevertheless, we will attempt in the next section to describe the control of crystal morphology exerted on HA crystallisation when precipitated from solution.

#### 4.1.2. Crystal shape control of HA precipitated from solutions

Several techniques have been used to synthesize HA from solution (wet synthesis) which include conventional chemical precipitation, solvothermal precipitation (it includes hydrothermal precipitation), emulsion, and sol–gel synthesis, among others.^30,31^ The common procedure in these wet synthesis implies the choice of a calcium and phosphate source which is solubilised in a chosen solvent and allowed to precipitate. Conventional chemical precipitation covers reactions at ambient pressure and up to the boiling temperature of the solvent. Solvothermal/hydrothermal syntheses are usually identified by the reaction of chemicals at elevated temperature (*i.e.* above boiling temperature) and pressure and are conducted in pressure vessels. Solvothermal/hydrothermal synthesis is widely performed in one or two step processes, and stands as a unique method to obtain high aspect ratio HA. This process yields highly crystalline products, whereas the morphology is highly dependent on the ratio between polar/apolar solvents used during synthesis, and hence, the vapour pressure achieved in the vessels, reactors, or autoclaves. HA nucleation is promoted *via* high temperatures, whereas the crystallite growth is controlled by the time of reaction and the surfactant or reverse micelle solution employed. These reagents offer an organized template guiding the growth of ordered crystals through several interactions as stereochemical, electrostatic or geometrical. An important parameter for high aspect ratio nanostructures is the pressure inside the reactor, governed by the vapour pressure of the solvents used, which in turn creates enough fluid convection and shearing stress for crystals to grow. However, fluid convection and shear stress can hamper the growth of highly oriented crystals such as nanowires when the vapour pressure is higher than the saturated vapour pressure of the solvents used.^32^ The sol–gel method involves precipitation on a 3D inorganic network of alkoxides. This network provides a template for mixing calcium and phosphorus precursors at a molecular level, which is capable of improving chemical homogeneity of the resulting HA. Sol–gel synthesis employ lower processing temperatures compared to solvo/hydrothermal methods, and allow for a tight control of chemical homogeneity, although they require post-calcination which can lead to structural changes, higher costs, and limited scalability. In the emulsion method instead, confined domains for precipitation are created by dispersion of two immiscible liquids stabilized with surfactants.

In general, it has been observed that HA crystals with hexagonal symmetry (*P*6_3_/*m*) when precipitated in the absence of templates (*e.g.*, organic molecules) grow along the *c*-axis direction exhibiting either plate-like or needle like morphology. The plate-like morphology is associated to the precipitation of metastable phases such as octacalcium phosphate (OCP) which is plate-shaped.^33^ The lower interfacial energy of OCP than that of HA is what drives OCP precipitation, which is followed by HA nucleation on top of it, preserving the plate shape (epitaxial growth).^34^ Now, without mediation of OCP, and under thermodynamic control, one would expect HA with hexagonal symmetry to grow either as needle-like/rod shape – if growth is favoured along the *c*-axis direction *i.e.*, piling up of hexagons-, or in the form of plates if the crystal grows in the *ab*-axis, by joining prismatic faces (Fig. 4). Which of these two possibilities prevails has been predicted by surface energy analyses of the different crystal faces.^35^ Studies by molecular dynamic simulations have shown that the (100) face has lower interfacial energy (324.8 mJ m^−2^) than the values of the (110) and (004) faces (698.6 and 1046.2 mJ m^−2^) respectively, which translates into decreasing surface area values of the synthesised HA crystals in the order of (100) > (110) > (001), supporting a needle-like/rod shape.^35^

Although under thermodynamic control the needle/rod form should prevail, many studies concerning the spontaneous precipitation of HA by conventional routes at both low and high supersaturation conditions have reported that crystallization remains under kinetic control and thus, mediated by the precipitation of metastable phases such as amorphous calcium phosphate (ACP) (under high pH), OCP (under neutral pH) and dicalcium phosphate dihydrate (DCPD) (under acidic pH) that would subsequently evolve to HA.^36^ The final morphology of the HA crystals may vary depending on the stabilisation of these intermediate phases. We earlier mentioned that precipitation of OCP as an intermediate phase can lead to the formation of HA crystals with plate-like morphology if HA grows epitaxially on OCP. However, OCP itself can transform into hexagonal rod-shaped HA crystals provided that a slow increase in pH from 4.4 until 6.7 is allowed.^37^ Similarly, ACP, considered as a metastable/unstable phase precursor, transforms to HA by an internal rearrangement process rather than by a dissolution–reprecipitation process, leading to the formation of needle-like crystals.^38^ The transformation of DCPD into HA requires prior dissolution of DCPD and no evidence of direct structural transformation from one phase to the other has been observed.^39^ To add further complexity into the crystallisation process, we need to add that the morphology of the grown crystals is highly influenced by the preparation conditions including the pH, temperature, and concentrations of the precursor species.

Most routes that produce high-aspect-ratio nanostructures are based on solvothermal reactions, as this method allows a wide range of useful working conditions, combining temperature and pressure. A wide range of crystal dimensions and shapes can be achieved by varying the nature, stoichiometry and concentration of reactants, temperature, pH, pressure and processing time (and additives) as reviewed in various works.^40,41^Table 1 summarises the main routes and the ranges of aspect ratio achieved, processes that are further described in the following paragraphs.

**Table 1:** Main routes and conditions to obtain high aspect ratio HA nanostructures precipitated from solutions (CCP: conventional chemical precipitation, HT: hydrothermal, ST: solvothermal; S: solvent; A: additive, AR: aspect ratio, HAR: high aspect ratio)

Synthesis	Ca, P_i_ precursor	Crystal growth control	Additives (A)/solvent (S)	Conditions	Range of AR	Crystal morphology	Comments	Ref.
CCP	Ca(NO_3_)_2_ 4H_2_O, (NH_4_)_2_HPO_4_	pH control with NH_4_OH or HNO_3_	^(S)^H_2_O	*T* = 50 to 200 °C, *t* = 20 to 100 h	n.s.	Rods to nanorods	Mixtures of HA/DCPA/OCP	42 and 43
CCP	CaCl_2_, Na_2_HPO_4_	Organic templates/micelles	^(S)^H_2_O, ^(A)^polysorbate, ^(A)^sorbitan monolaurate, ^(A)^sodium polyacrylate, ^(A)^polydiallyldimethylammonium chloride	*T* = 25 to 60 °C	n.s.	Neuron-like	Neuron-like structures assembled into clusters with growing fibres of 1 to 2 μm length, with few nm of diameter	44
HT	CaCl_2_, Na_5_P_3_O_10_	P_i_ concentration, saturation index (SI)	^(S)^H_2_O, ^(A)^glutamic acid, ^(A)^(NH_4_)_2_CO	*T* = 180 °C, *t* = 10 h	n.s.	Nanorods (SI > 0), prism-like nanorods (SI < 0)	Nanorods, hexagonal prisms, microspheres, and flowerlike structures grow governed by SI	45
HT	Ca(NO_3_)_2_ 4H_2_O, H_3_PO4	pH control with NH_4_OH	^(S)^CH_3_CH_2_OH	*T* = 140 to 220 °C, *t* = 1 to 12 h	2.3 to 4.9	Nanorods	↑AR at ↓pH, pH 8–10–12	46
HT	Ca(NO_3_)_2_ 4H_2_O, (NH_4_)_2_HPO_4_	pH control with (NH_4_)_2_CO	^(S)^H_2_O	*T* = 90 or 200 °C, *t* = 60 h	3 to 24	Nanorods	↑AR at ↓pH, ↑AR at ↑T	47
HT	Ca(NO_3_)_2_ 4H_2_O, (NH_4_)_2_HPO_4_	Solvent and pH control with NH_4_OH	^(S)^CH_3_CH_2_OH, ^(S)^CH_3_OH, ^(S)^(CH_3_)_2_CHOH	*T* = 150 °C, *t* = 24 h	n.s.	Nanorods, hexagonal rods, needle	Variation in concentration with CH_3_CH_2_OH yields HAR	48
ST	Ca(NO_3_)_2_ 4H_2_O, NH_4_H_2_PO_4_	Oleate template	^(S)^H_2_O, ^(S)^CH_3_CH_2_OH	*T* = 120 °C, *P* = 7 bar, *t* = 20 to 96 h	100	Nanowires	Nanowires of 37 nm in diameter and 3.7 μm in length	49
ST	CaCl_2_, NaH_2_PO_4_ 2H_2_O	Oleate template	^(S)^H_2_O, ^(S)^CH_3_OH, ^(A)^oleic acid	*T* = 180 °C, *t* = 40 h	7 to 10 000	Nanowires	Lengths > 100 μm	50
ST	CaCl_2_, NaH_2_PO_4_ 2H_2_O	Organic template, P_i_ concentration	^(S)^H_2_O, ^(S)^CH_3_CH_2_OH, ^(A)^stearic acid, ^(A)^palmitic acid	*T* = 180 °C, *t* = 24 h	>1000	Nanofibres	Different sources of P_i_ precursors: NaPO_3_/Na_5_P_3_O_10_/Na_2_HPO_4_/Na_2_H_2_P_2_O_7_	51
ST	CaCl_2_, NaH_2_PO_4_ 2H_2_O	Oleate template	^(S)^H_2_O	*T* = 200 °C, *t* = 36 h	65 to >1000	Nanowires	1D to 2D assembled nanowires after immersion in CH_3_CH_2_OH	18
ST	CaCl_2_, NaH_2_PO_4_	Oleate template and alcohols	^(S)^CH_3_CH_2_OH, ^(S)^CH_3_OH, propanol/butanol/pentanol/hexanol, ^(A)^Oleic acid	*T* = 180 °C, *t* = 18 h	100 to 1000	Nanowires	Lengths from 100 μm to mm	52
ST	CaCl_2_	P_i_ concentration and source	^(A)^Oleic acid	*T* = 180 °C, *t* = 25 h	10 to 1000	Nanorods, nanowires, nanofibres	Different sources of P_i_ precursors: NaH_2_PO_4_/Na_2_HPO_4_/Na_3_PO_4_/Na_5_P_3_O_10_/Na_4_P_2_O_7_	53
ST	CaCl_2_, NaH_2_PO_4_ 2H_2_O	Oleate template	^(S)^CH_3_CH_2_OH, ^(A)^oleic acid	*T* = 180 °C, *t* = 5 to 23 h	>100	Nanofibres	Self-assembly of nanofibers upon immersion in CH_3_CH_2_OH, yields a fibre entanglement 28 mm long	54 and 55
ST	CaCl_2_, NaH_2_PO_4_ 2H_2_O	Oleate template	^(S)^H_2_O, ^(S)^CH_3_OH, ^(A)^oleic acid	*T* = 180 °C, *t* = 24 h	>100	Nanofibres	Post-alignment of fibres using ice-templating	56

Supersaturation, for example, is a key parameter in the design of HA crystals with the desired shape. It has been shown that, in both hydrothermal and conventional precipitation routes, larger crystals develop at low supersaturations, consistent with the fact that crystal growth dominates over nucleation, whereas an increase in supersaturation favours nucleation over crystal growth, resulting in the formation of smaller crystals.^45,57^ Two other major parameters affecting the morphology and structural characteristics of the precipitating HA are temperature and pH. The aspect ratio of hydrothermally precipitated crystals has been reported to steeply increase with decreasing the pH value by favouring anisotropic growth along the *c*-axis at low pH. This effect has been explained by the ability of different ionic species existing in solution for a given pH to alter the structure of the ionic ligand, resulting in different growth rates along the *a*, *b* and *c* axes of the HA crystal.^36,46^ However, if the pH becomes too low (<4), the stabilisation of metastables phases comes into play leading to more complicated shapes.^21,46,47^ Similarly, an increase in the temperature increases the *c*-axis length of the crystal and improves crystallinity and stoichiometry.^21,47^ The increase in crystallinity is further favoured at high pH owing to the suppression of the competing HPO_4_^2−^ and H_2_PO_4_^−^ in favour of PO_4_^3−^. Unfortunately, not all authors report these same trends which implies that hydrothermal nucleation and crystallisation are an interplay of many interdependent parameters which makes interpretation of the results difficult.

The incorporation of organic solvents in solvothermal processes is common. In fact, the use of solvents or solvent mixtures is often employed to alter solvent–solute interactions at the molecular level to favour specific interactions with the nuclei. This allows a reduction in the interfacial energy improving the rate of crystal growth in specific directions.^58,59^ In a recent work by Sans *et al.* they explored the synthesis of HA with different shapes and sizes varying different parameters including the use of non-aqueous solvents.^48^ Moreover, Nagata was able to show a change in the crystal morphology from plate-like to rod-like depending on the amount of methanol added.^58^

But perhaps one of the preferred strategies for controlling crystal morphologies is through the use of additives (ions, surfactants, organic molecules, peptides, proteins, *etc.*) which can act by adsorbing on the precipitating nuclei/crystal (soft-templates) and/or by controlling the availability/concentration of ions *via* ion-chelation.

When additives are added at a low concentration -to not quench crystal growth- and in a thermodynamic regime to allow layer-by-layer growth, they can selectively adsorb to certain faces of the crystal reducing their surface energy and thus their growth rate. In such circumstances, the crystal grows predominantly on the uncovered faces following Wulff's rule.^28^ Alternatively, in syntheses performed under kinetic control *i.e.*, with a high concentration of reagents and additives, supersaturation becomes so high that induces the formation of many nuclei that can aggregate into larger crystals. This aggregation can be suppressed if a high concentration of a stabilizing additive is added, resulting in the creation of a dispersion of nanoparticles. But interestingly, if the additive is adsorbed only on specific faces of the nanocrystal, the nanocrystals can self-assemble so that they adopt a mutual orientation in a crystallographic record, merging.^28^ An example of this later case is the formation of 1D ultralong HA nanofibers by self-assembly of small building blocks partially stabilised with molecules such as sodium oleate.^50^ This case will be explained in more detail in the following section.

Another scenario worth mentioning is when the additive binds to metastable phases such as amorphous calcium phosphate (ACP) rather than to a crystalline nucleus. Amorphous phases, being unordered, and therefore isotropic, can be moulded into any shape, allowing to obtain precipitates with complex morphologies. This is the case, for instance, of the precipitation of amorphous calcium–phosphate neuron-like structures with the help of organic additives such as surfactants, small organic molecules and peptides.^44,60^ In general, ACP is a highly unstable phase that hydrolyses almost instantaneously to HA. However, adsorption of organic molecules onto ACP not only shields this phase from evolving, but also aids in the self-assembly of complex and “stable” structures.

But additives can also act by sequestering the ionic species necessary for precipitation. This is the case, for example, with the calcium chelating agent EDTA (ethylenediaminetetraacetic acid). After chelation, the concentration of free calcium ions in solution decreases substantially, leading to the precipitation of lesser and smaller nuclei. During hydrothermal treatment, the calcium ions are slowly released, and each individual nucleus will grow in a more controlled manner to form a distinct crystal.^21^

Despite the use of additives has been widely exploited for the stabilisation/control of many shapes and sizes of HA crystals, pinpointing the effect of the additive on the final morphology of the crystal is not trivial due to the multiple interplay between parameters during the synthesis reaction.

### 4.2. Synthesis and processing of bulk high-aspect-ratio hydroxyapatite materials

#### 4.2.1. Nanofibrous ceramics using soft templates

The design of HA ceramics as ultralong 1D nanofibers is a promising strategy to overcome the inherent brittleness of ceramics. Indeed, 1D ceramic materials synthesised in the form of fibres have extremely large aspect ratio and good fibre continuity. This has been reported to provide them with the potential to tolerate large bending deformations by axial accumulation of small local deformations due to its ability to absorb and dissipate stresses through large axial deformations.^61^ These fibres assembled into bendable membranes and/or flexible scaffolds may revolutionize *e.g.* the biomedical field by providing a new class of materials that would combine the flexibility acquired by the ceramic fibre with the excellent biocompatibility, bioactivity and osteoconductivity of HA.

**Fig. 6:**
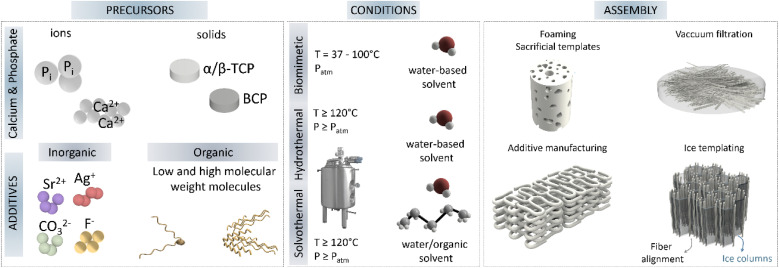
Main synthesis routes for nanostructure HA, highlighting the precursor sources often used, together with common additives, the conditions frequently used to synthesise high aspect ratio HA; and finally, the common 3D assembly strategies to obtain 3D ceramics (TCP-tricalcium phosphate, BCP-biphasic calcium phosphate).

Despite there have been many attempts to synthesize 1D-HA ultralong nanofibers using different synthesis methods,^31^ one rather stablished method is the one that involves the formation of calcium oleate (or similar molecules) as precursor (Fig. 6).^18,50–54,62^ Oleate has the double purpose of sequestering calcium forming calcium oleate complexes and to adsorb onto the prismatic faces of the growing HA, rich in calcium, directing fibre growth. The mechanism underlying the formation of the ultralong 1D HA fibres can be explained following the same arguments as for the precipitation of other 1D fibres such as BaSO_4_. Thus, when crystallisation starts, small building units begin to precipitate where oleate selectively coats the (100) crystal faces of HA rich in calcium thus transforming them into low-energy surfaces (adsorbed polymer) compared to the high-energy faces *i.e.*, the (001) faces, which have no adsorbed oleate. Subsequently, these small building units self-assemble to reduce their surface energy by means of an oriented attachment mechanism, where equal high-energy (001) surfaces of two neighbouring building units crystallographically combine and fuse to form a single crystal. By subsequent fusion of these small building units ultralong fibres can be formed. These syntheses when using oleate as precursor need to be performed in high pressure reactors to achieve a slow and sustain release of calcium from the Ca–oleate complex. The resulting nanofibers are crystalline and single phase.

Importantly, the post-assembly of these ultralong nanofibers is not trivial, and different consolidation methods have been applied to obtain functional end-products. The conversion of these ultralong nanofibers into 2D membranes and 3D scaffolds can be achieved by different strategies. Thus, membranes can be easily obtained by vacuum filtration of fibre slurries, while porous 3D scaffolds have been prepared by ice templating by freezing aqueous slurries containing the ultralong 1D nanofibers and subsequent ice sublimation.^56,63,64^ An important post-assembly process for ultralong nanofibers mainly produced *via* solvothermal synthesis, takes advantage of the presence of oleate molecules and their interactions with solvents. In such cases it is possible to obtain an alignment of fibres into bundles or nanoropes by immersing the HA nanowire dispersion into ethanol baths, usually with the help of an injection mechanism.^50,54^ Described as a surface-induced self-assembly process, the alkyl chains from oleic acid adsorbed onto HA nanowires/nanofibers diffuse into the solution, resulting in the segregation of the nanowires from the solution and thus enabling their assembly. Moreover, although not yet described for 1D-HA nanofibers, 3D printing of scaffolds by extrusion of aligned fibres may soon be possible, as already described for nanomicellar fibrils in the work of Rajasekharan *et al.*^65^ The use of extrusion printing tools, in addition to allowing the construction of controlled architectures, may also result in fibre alignment due to shear forces applied during extrusion along the flow direction, which will impact the mechanical properties of the final scaffold.

A key aspect of these highly ordered structures is their superior mechanical properties. For instance, 1D HA nanowires assembled into 2D interwoven networks can solely increase the tensile strength by 5 MPa without the addition of any reinforcement filler or binder, compared to their 1D arrangement.^18^ In contrast, HA nanowires assembled into nanoropes can withstand stresses up to 100 MPa, with relative deformations up to 3%, very uncommon for fully ceramic materials.^49^ Higher deformations, up to 30%, can furtherly be achieved by combining HA nanowires with only 2 wt% of polymers (chitosan),^62^ or reversible deformations up to 100 cycles in combination with polyimides, demonstrating an elastic behaviour, likewise uncommon for ceramic materials.^66^

#### 4.2.2. Hard template based nanostructured cements and ceramics

Another strategy to construct bulk materials with controlled high-aspect-ratio surfaces is to take advantage of cementitious reactions based on the hydrolysis of reactive calcium phosphate materials, such as alpha-tricalcium phosphate (α-TCP). These reactions involving solid precursors can be considered as “hard template” synthesis as the reaction takes place on the precursor surface.^41^ This strategy basically exploits the conversion of an unstable solid (*e.g.* α-TCP) into a stable solid such as HA by dissolution–precipitation reactions and allows obtaining bulk samples with nanostructured topographies. By controlling the dissolution rate of the solid, the surrounding environment and the incorporation of additives (soft templates) a wealth of crystal morphologies can be precipitated.

As mentioned, different aspects of the reaction can be tuned to control the morphology of the precipitating crystals. One first parameter is to alter the solubility of the solid template as it will affect supersaturation and therefore nucleation and crystal growth. In the works of Ginebra *et al.* and Espanol *et al.* α-TCP milled to different particle sizes was hydrolysed resulting in microstructures consisting in an entangled network of larger and less dense plate-like crystals when large α-TCP was used and in an entangled network of smaller and very dense needles when smaller α-TCP was used.^67,68^ This different behaviour is explained by the different degree of supersaturation attained from particle dissolution. The higher degree of supersaturation achieved with smaller α-TCP particles favoured formation of many nuclei, while lower supersaturation yielded fewer nuclei but favoured their growth.

Changing the hydrolysis reaction of α-TCP from body temperature to hydrothermal conditions (autoclaving at 121 °C) changed the crystalline morphology from plate-shaped crystals to needle-shaped crystals of higher aspect ratio, respectively. In addition, the higher reaction energy of the autoclaving process favoured the nucleation of apatite with higher crystallinity.^69^ In another work by Diez-Escudero *et al.* during the hydrolysis reaction, carbonate ions were incorporated demonstrating the potential of additives, carbonate ions in this case, to control morphogenesis. The presence of these ions substantially retarded the hydrolysis reaction and led to the stabilization of small plate-like crystals, due to the distortion that carbonate ions cause in the HA crystal lattice.^70^

One of the advantages of working with hard templates such as α-TCP, which are capable of undergoing hydrolysis reactions, is that they can be easily consolidated into bulk forms. In fact, α-TCP in the form of powder (particles), when mixed with an appropriate proportion of water, has the ability to consolidate into a hard-bulk material due to the interlocking of HA crystals between the particles (cementitious reaction). α-TCP powder can also be extrusion printed with the help of polymers such as poloxamers or other polymeric carriers and subsequently hydrolysed to form rigid 3D printed scaffolds while solubilising the organic carrier.^71,72^ The combination of additive manufacturing technologies with different hardening treatments, involving either conventional hydrolysis at body temperature and atmospheric pressure (biomimetic conditions), or hydrothermal treatments, allow controlling the morphology of the resulting crystals and their aspect ratio. Thus, it has been shown that the consolidation of 3D-printed α-TCP structures using a hydrothermal process results in a high-aspect-ratio needle-like HA crystals elongated along the *c*-axis, in contrast to plate-like morphology obtained when the consolidation is performed at 37 °C and atmospheric pressure.^69^

Likewise, other calcium phosphates can be used as hard templates. β-TCP particles or biphasic calcium phosphates (BCP) particles can be assembled into bulk samples by sintering and subsequently submitted to hydrothermal treatments to create the desired nano/micro texture *via* dissolution/recrystallisation reactions.^73^

### 4.3. High-aspect-ratio nanostructured hydroxyapatite coatings

HA coatings are frequent in the biomedical field with the purpose of providing a more bone-like structure to metallic implants. In addition, HA based coatings can improve corrosion resistance to metals.^74–77^ The synthesis of high-aspect-ratio HA nanostructured coatings is normally achieved by precipitation methods, either biomimetic or hydrothermal, that can be combined with microwave or electrical stimulation.

A microwave assisted method was proposed to synthesize controlled HA nanostructured coatings on Mg alloy. The microwave radiation time helped controlling HA nucleation and growth by disrupting the electric double layers near hydroxyls groups in HA, promoting a *c*-axis oriented crystal growth as the radiation time increased, and aligned perpendicular to the substrate. Closer to the Mg alloy substrate, Mg ions were incorporated into the HA lattice resulting in plate-like crystals, while far from the substrate surface, HA crystals had a higher aspect ratio, with a whisker like morphology.^77^

Similar perpendicular oriented coating on Ti alloy (Ti6Al4V) with highly (0 0 2) plane-oriented HA nanorods were obtained using magnetic bioglass as sacrificial template during hydrothermal synthesis (12 h to 3 days at 120 °C). The magnetic nanoparticles (Fe_3_O_4_) incorporated into HA had a strong effect on crystal morphology together with the reaction time; shorter hydrothermal treatment time (24 h) resulted in perpendicular nanorods which transformed into plates after 3 days of treatment; however, in the absence of magnetic nanoparticles, the same long treatment derived into grain blocks rather than nanorods.^78^ A combination of microarc oxidation and hydrothermal treatment was also used to obtain bilayer coatings of nanorod HA and MgO.^74^ HA nanorods were obtained *via* hydrothermal treatment in presence of anionic EDTA for 2 h to 24 h on the microarc oxidized Mg substrates, obtaining higher aspect ratio nanorods with longer hydrothermal treatment time (24 h).

By using biomimetic precipitation, the shape of HA nanostructures can be easily modified using doping elements. The incorporation of strontium, fluoride or silicate into ionic saturated solutions for HA precipitation has resulted in varied nanostructured HA morphologies from spheres, nanowhiskers or plate-like crystals, respectively.^79^

Electrodeposition by means of inducing HA precipitation *via* electrical stimulation has been applied to obtain multi-doped high-aspect-ratio HA.^75^ Nanofluorhydroxyapatite co-doped with Ag and Sr was electrodeposited in one single step onto titanium substrates to exert antibacterial properties from silver release, while balancing the cytotoxicity from silver to mammalian cells with strontium. Pure HA and fluorhydroxyapatite HA resulted in nanowire structures, with fluorhydroxyapatite resulting in wider wires and more compact, whereas the incorporation of strontium yielded packed nanorods of 50 to 100 nm thick. Similar electrodeposited coatings based on nanostructured HA were obtained on anodised titanium to obtain TiO_2_ nanotubes. Silver-doped HA and calcium silicate mixtures were later electrodeposited onto the TiO_2_ nanotubes with similar application to improve antibacterial properties *via* silver ion release and improve cellular response *via* silicon ions. In this case, the high-aspect-ratio nanowires were confined into the TiO_2_ nanotubes.^76^ Finally, electrospun fibres combining silver doped HA wires with polymers (polylactic acid based) have been used as coating with concentrations up to 40 wt% HA. Although the high-aspect-ratio in this case was not inherent to the ceramic HA, but its combination with polylactic acid.^80^ Although not extensively exploited, electrospinning of polymeric materials in combination with high-aspect-ratio HA can help overcoming the mechanical limitations during composite fibres formation.^81^

## 5. Functional properties and applications of high-aspect-ratio hydroxyapatite materials

### 5.1. Effect of the aspect ratio on physical, chemical, and mechanical properties of hydroxyapatite

High-aspect-ratio nanostructured materials possess unique properties which make them appealing for a plethora of applications in nanotechnology, material science, and biomedical engineering. The high-aspect-ratio morphology is linked to an increased flexibility of the crystals, and to a higher specific surface area. Moreover, the spaces between the long crystals allow obtaining highly porous materials, and the preferential orientation of the crystal growth results in specific elemental or molecular interactions with the atoms arranged in the exposed crystal planes. Moreover, depending on their configuration, high-aspect-ratio nanostructures can also be highly anisotropic in their optical, magnetic, electrical, and chemical properties, when single-direction aligned crystal growth occurs during synthesis, and the growth of a particular crystal plane is favoured over others.^82^ All these properties, combined with the intrinsic properties of HA, including the hospitable nature of its structure that allows incorporation of a wide variety of ions, the high adsorption capacity, and the good thermal and chemical stability, renders high-aspect-ratio HA materials highly attractive in fields as varied as energy, sustainability or biomedicine, which will be described in detail in the following sections.

**Fig. 7:**
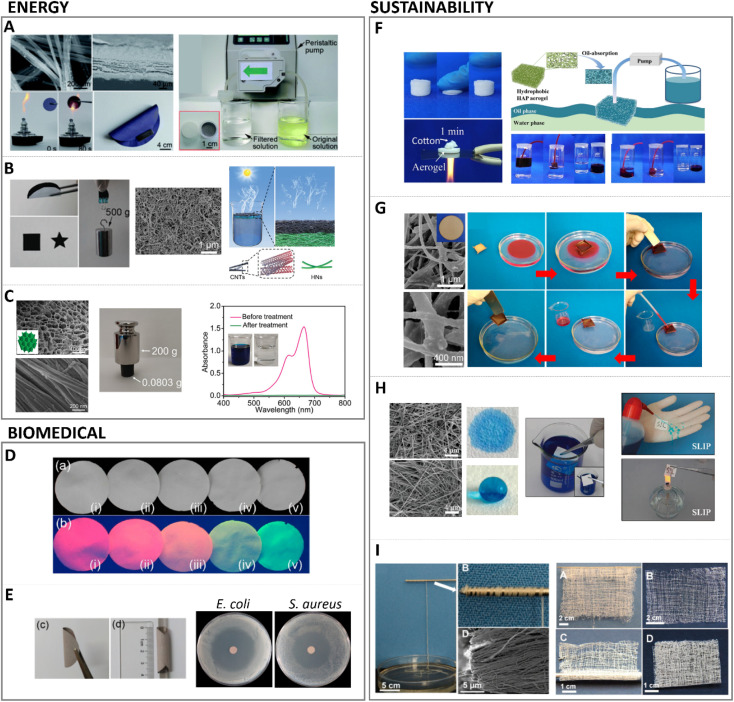
Main applications of high aspect ratio nanostructured HA. Energy applications for (A) catalytic fire-resistant paper (adapted with permission from ref. 83, copyright 2018 Royal Society of Chemistry); (B) high strength aerogel for energy driven water evaporation (adapted with permission from ref. 84, copyright 2018 Wiley), and (C) tree inspired aerogel for catalytic treatment of organic dyes (adapted with permission from ref. 56, copyright 2021 Wiley). Biomedical applications include (D) photoluminescent biomedical paper (adapted with permission from ref. 95, copyright 2017 American Chemical Society), and (E) antibacterial biomedical paper loaded with silver and antibiotics (adapted with permission from ref. 93, copyright 2017 American Chemical Society). Sustainability application of high aspect ratio illustrating (F) flexible aerogel for water–oil separation (adapted with permission from ref. 86, copyright 2018 American Chemical Society), (G) magnetic paper for on demand motion in oil filtration (adapted with permission from ref. 96, copyright 2018 American Chemical Society), (H) tuneable hydrophilic/hydrophobic synthetic fire resistant paper (adapted with permission from ref. 94, copyright 2016 American Chemical Society), and (I) large scale production of HA nanowires for flexible textiles (adapted with permission from ref. 50, copyright 2016 American Chemical Society).

Energy applications of HA as catalyst have been developed exploiting its ability to allow cationic and anionic substitutions in its structure. The use of high-aspect-ratio nanostructured HA increases the surface area available to allocate such cationic and anionic substitutions. Further control of HA crystal growth, such as highly *c*-axis oriented high-aspect-ratio nanostructured HA, enables the selective substitution of the elements exposed in that plane, improving its catalytic efficiency. The design of materials with a controlled porosity or possessing hierarchical texture have aroused great interest due to the possibilities it opens up in heterogeneous catalysis.^2^ Aerogels, membranes and synthetic paper are the proposed forms for catalytic applications. Additionally, high aspect ratio HA can be combined with natural polymers such as chitosan to improve its mechanical properties, while enhanced catalytic activity can be achieved by incorporating for instance, Au or Pd nanoparticles along the highly oriented HA fibres. A synthetic paper consisting of ultralong HA nanowires combined with Au nanoparticles exhibited high catalytic activity for continuous flow reduction of 4-nitrophenol. The thickness of the paper membrane dictated the conversion efficiency of 4-nitrophenol to 4-aminophenol, where thicknesses of 53 to 127 μm resulted in 95 to 100% conversion efficiency. This was ascribed to the increased surface area available for interactions with the Au nanoparticles immobilised onto the HA nanowires surface (Fig. 7A).^83^ A similar photocatalytic synthetic paper was obtained by combining ultralong HA nanowires with carbon nanotubes. This synthetic paper exhibited high light absorbability and photothermal conversion applied to solar energy-driven water evaporation (Fig. 7B).^84^ Moreover, due to the good thermal stability of HA, it acted as thermal insulator providing stability under high temperature conditions, while the high-aspect-ratio contributed to form a porous network of intertwined fibers and bundles which improved the water transportation path and evaporation surface.

Further catalytic activity of HA has been investigated by combination with Pd.^56,85^ Immobilised Pd clusters have shown high efficiency in alcohol oxidation, with higher reactivity towards benzylic and allylic ones, over aliphatic alcohols.^85^ These findings fuelled the use of HA ultralong nanowires combined with Pd nanoparticles as nanocatalyst for continuous flow catalysis of, for instance, methylene blue.^56^ In this approach, studied by the same group who developed the Au doped HA synthetic paper aforementioned,^83^ chitosan was also added to the HA nanowires, obtaining a flexible aerogel.^56^ Additionally, instead of applying vacuum filtration, unidirectional freeze drying was used to build this aerogel/membrane, resulting in a structure with vertically aligned channels similar to tree channel transportation (Fig. 7C).

Energy application materials such as thermal insulating materials or aerogels are often obtained or fabricated by high cost efficiency methods, involving organic components which are highly flammable or thermally unstable, or by using high time and cost consuming techniques such as critical point drying. In this regard, HA represents a low toxicity and fabrication costs alternative material, and highly recyclable due to its biocompatible nature.^86^

Some of the materials with application in the energy field have also found use in sustainability approaches such as water purification, and pollution treatment, synthetic paper or sustainable textiles. HA has been investigated as adsorbent for removal of wastewater metals and inorganic elements, permeable reactive barriers in nuclear waste, or the removal of organic waste such as dyes generated in textile industries.^1^ Water, air or oil filtration membranes or aerogels require lightweight materials with low density, high porosity, and high surface area resulting in low thermal conductivity and low acoustic propagation speed, properties that are fulfilled by high-aspect-ratio HA, together with the low fabrication costs and recyclable nature of HA compared to conventional materials.^86^ Additionally, HA has great affinity for substituting and immobilizing cations and anions in its structure.

Beyond the hospitable lattice structure of HA to substitute elements, the morphology of the HA crystals has shown to modify the metal ion removal efficiency. Higher aspect ratio HA nanorods exhibited higher efficiency removing Pb, Cu, Mn and Zn cations, compared to lower aspect ratio plate-like crystals and spherical particles.^87^ Previous research has shown that the affinity, for instance for cations, is highly dependent on the radii of the substituting element^88^ (higher ionic radii than Ca ions are more likely to be incorporated into HA lattice, such is the case of Pb compared to Zn); lower crystallinity HA has also effects on its adsorption capacity, due to higher specific surface area in low crystalline HA.^89^ Thus, the use of high-aspect-ratio HA with low crystallinity offers an improvement window in the adsorption capacity and the efficiency in pollution treatments. Anions as fluoride have also been successfully separated from water waste using HA nanowires, assembled into membranes using simple vacuum filtration. The high-aspect-ratio of HA nanowires alone outperformed the adsorption capacities of other HA types such as biogenic apatite, nanosized apatites or composite HA (chitin–HA or cellulose–HA membranes), with adsorption capacities of 40 mg g^−1^, and water flow up to 350 L m^−2^.^90^ Enhanced adsorption capacity of high-aspect-ratio HA nanostructures has been explored by combination with other adsorbents such as limestone to improve the liquid retention and promote the adsorption mechanism of HA.^91^

Similar membranes based on HA nanowires have been obtained by combination with cellulose acetate, and further doped with Ag_3_PO_4_ to impart antibacterial properties in wastewater filtration.^92^ Similar biomedical papers have been produced purely based on HA nanowires, and combined with antibiotics and silver nanoparticles for enhanced antibacterial properties (Fig. 7E).^93^

The versatile hydrophilicity of HA nanowires, tuneable upon modification of the washing procedure in the solvothermal synthesis for instance, has found applications also in water–oil separation and air filtration. HA nanowires aerogels were obtained by solvothermal method followed by freeze drying. These aerogels were later washed with ethanol and water, or ethanol–sodium–hydroxide–water to yield highly hydrophobic or hydrophilic aerogels, respectively (Fig. 7H).^94^ Similar hydrophobic aerogels demonstrated to effectively filtrate particulate matter in polluted air, and adsorbed oils and organic solvents from water (Fig. 7F).^86^ Importantly, the high-aspect-ratio of the HA nanowires yielded elastic aerogels which could be squeezed, for instance, to remove the adsorbed organic solvents, allowing for desorption and membrane regeneration, or to shape the aerogels into different filtration devices without fracture. Furthermore, the hydrophilic aerogel exhibited very low thermal conductivity and fire resistance capacity. Similarly, membranes based on HA nanowires assembled by suction filtration also demonstrated this dual hydrophilicity, fire resistant and liquid repellence capacity, water–oil filtration ability. In addition, these membranes could be used as synthetic paper reducing wood and resources consumption, with water and fire proof properties, and anticounterfeiting properties upon rare earth elements doping (Fig. 7D).^94,95^ Moreover, magnetic properties were implemented in HA nanowire-based synthetic paper by incorporating Fe_3_O_4_ particles. After synthesis of HA nanowires, they were decorated with magnetic particles, vacuum filtered and coated with polydimethylsiloxane (PDMS), resulting in a flexible magnetic paper. In addition to the mentioned water–oil separation properties of these membranes/papers, the magnetic nature of this synthetic paper was employed for recovery of the filtered oil, adsorbed on the paper, under magnetically driven manipulation (Fig. 7G).^96^

Owing to the outstanding flexibility and strength of high-aspect-ratio HA nanowires, stretchable membranes and fibres have been synthesised with application as textiles.^50^ Importantly, the assembly of HA ultralong wires with aspect ratio larger than 10 000 was performed at room temperature by maintaining the HA nanowires dispersed in ethanol, avoiding post-processing methods for assembly as filtration or freeze-drying. This enabled the manipulation of HA nanowires dispersion as nanoropes, capable of withstanding 500 g of weight (36 twisted nanoropes). The fibers or nanoropes showed tensile strengths of almost 100 MPa (Fig. 7I). Furthermore, the good thermal stability of HA was preserved, providing fireproof textiles.

Last but not least, high-aspect-ratio nanostructured HA has greatly impacted the biomedical field with new applications such as biosensing, bioimaging, or as improved biomaterials. High-aspect-ratio-based structures have been doped with rare earth elements (lanthanides) and used as chemical catalysts for their particular optical and magnetic properties due to their anisotropic growth, and later alignment. Increasing the aspect ratio of synthesised HA results in enhanced adsorption rates, useful for delivery of contrast agents or medications. Usually, HA is chosen for its ability to substitute hydroxyls OH^−^ with, for instance, fluorides F^−^ which yield low vibrational energy materials, favouring rare-earth fluorescent transition doping with rare-earth elements.^97^ Noteworthy, depending on the ionic radii of the lanthanide doping element, the growth behaviour of HA crystals varies. Additionally, these shapes can be further modified by balancing the ratio between cationic/anionic surfactants. For instance, using oleic acid and oleylamine during synthesis results in various crystal shapes from nanorods to rice-like crystals. If only an anionic surfactant is used (oleic acid), the same crystals convert to ultrathin nanowires with aspect ratio larger than 1500 (3–4 nm diam. 5–10 μm length).^97^ These photoluminescent properties upon rare earth element doping HA have been applied to biomedical papers,^96^ similar to previously discussed energy and sustainability applications. HA nanorods co-doped, *via* ion exchange, with Yb^3+^ and Ho^3+^, or alternatively co-doped with Eu^3+^ and Gd^3+^,^98,99^ have been successfully used as multiplexed labelling probes for bioimaging owing to the upconversion capacity (*i.e.* ability to absorb two or more low-energy photons and radiate a high-energy photon) of lanthanides and their tuneable colour output from ultraviolet to near infrared emissions under infrared radiation.^100^

Moreover, when high-aspect-ratio HA are assembled in a controlled spatial array or structure, they can have important properties beneficial in sensors or biosensors. The good biocompatibility of HA, combined with its high adsorption enhanced when high-aspect-ratio nanostructures are present, makes HA an excellent candidate for sensing gas, liquids or specific molecules such as peroxidase^101^ or cyanide.^102^ The application of high-aspect-ratio HA into biosensors is blooming and principle supported by previous studies using HA nanoparticles in sensors. For instance, a tube-like Au modified HA was investigated as a gas sensor for detection of NH_3_ (ref. 103) resulting in an improvement on sensitivity due to the role of hydrogen and hydroxyls retaining water which later help adsorbing ammonia. Furthermore, the tube-like structure of HA increased the surface area for NH_3_ interaction, improving physisorption. The combination of HA with gold nanoparticles resulted in a higher electron density.^103^ Usually biosensors rely on the detection of an electrochemical signal which requires a conductive electrode or transducer. Thus, common strategies have explored the functionalization of conductive electrodes based on gold^104^ or carbon with HA.^105^ In such applications, due to the biocompatibility of HA and its multiple interactions with enzymes or proteins *via* calcium and phosphate groups exposed in its crystal planes, the retention of the targeted molecules is enhanced.^101,106^ Although the used of high-aspect-ratio HA in sensors is at its infancy, once implemented, future improvements can be achieved due to the larger adsorption available area inherent to these surfaces.

One important property of HA nanostructures, uniquely inherited from their high aspect ratio, is their high flexibility. Owing to the almost 1D nature of high-aspect-ratio structures, their mechanical properties can be tuned by, for example, controlling the number of nanofibers, their spacing and interlocking, and further improved by combination with polymers such as chitosan or collagen. When combined with polymers, the length of the HA nanostructures has shown to be key in mechanically reinforcing the paper, regardless of the application.^107,108^ Sun *et al.* have intensively developed different co-loaded HA-based papers for applications such as biomedical paper decorated with Mg,^109^ Zn and combined with chitosan^110^ or collagen.^111^ Similarly, antibacterial properties have been achieved by means of silver incorporation for biofunctional paper applications.^93,112^ Other combinations with high-aspect-ratio HA with unique mechanical properties include carbon nanotubes,^84^ silver nanoparticles and antibiotics for water purification and antibacterial paper,^84,112^ or glass fibers as insulating materials in fiber-optic cables.^113^

### 5.2. Biological interaction of high-aspect-ratio HA: proteins, cells, and tissues

A few years ago, the terminology of metamaterials as applied to biological interactions of nanostructured surfaces was coined.^114^ A plethora of studies have focused their attention on the study and characterization of the interaction of proteins, cells and tissues with materials at the nanoscale. The advances in nanofabrication and characterization techniques have also boosted our scientific knowledge. The ability of cells to sense and adapt to the spatial environment, for both eukaryotic and prokaryotic cells, offers an application window for nanostructured surfaces. With regards to the importance of crystal conformation, it has been shown that cellular interactions with HA crystals also depend on crystal orientation. This is highly relevant also for the interaction with biomolecules or proteins, where preferentially oriented crystals can result in particular immobilization of higher affinity toward specific molecules.

#### 5.2.1. Protein interactions with high-aspect-ratio hydroxyapatite

The specific interaction of HA with some proteins or biomolecules, due to calcium, phosphate and hydroxyl exposure on the crystal faces, is the basis for the development of biosensors^105^ where its combination with conductive electrodes has been proven to increase the immobilization of proteins/enzymes (a-fetoprotein,^104^ protease β-protease,^115^ glucose/glucose oxidase^116,117^). In a biological setting, protein interaction with materials is crucial for later cell attachment, growth and differentiation, which also applies to tissue formation. These protein interactions have been particularly efficient in bone regenerative strategies.^118^ Accelerated by the development of proteomics,^119,120^ the preferential adsorption of certain proteins related to the nanostructure of HA has been studied, and the role of HA physical features in modulating protein recruitment, immobilization, and subsequent release has been explored.^121^

**Fig. 8:**
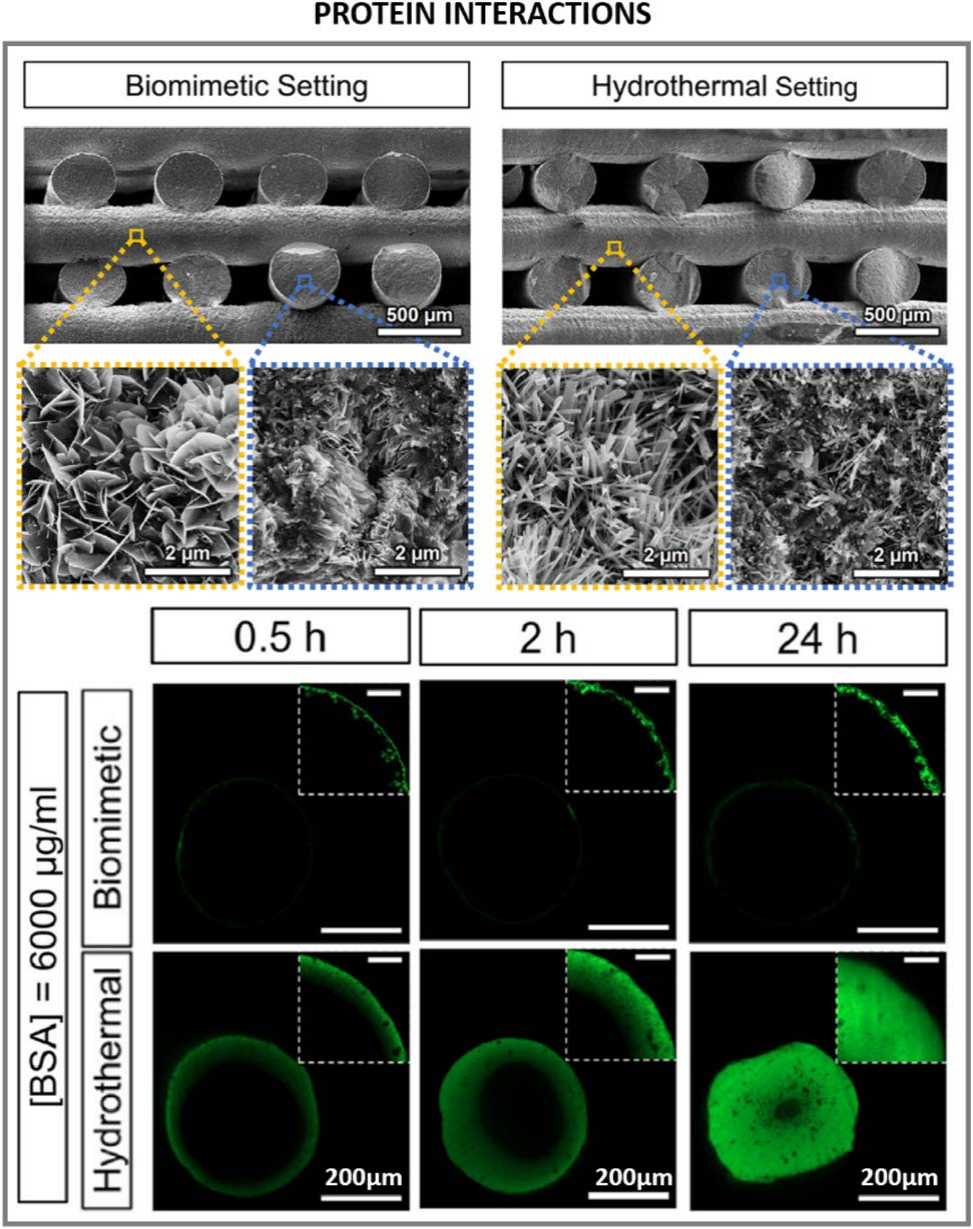
Biomedical applications and implications of high aspect ratio nanostructured HA in protein interactions with high aspect ratio HA (adapted with permission from ref. 69, copyright 2021 Elsevier).

Interestingly, it has been shown that high-aspect-ratio needle-like structures can yield three times higher protein adsorption and immobilization compared to plate-like structures, correlated to the increase in specific surface area, the presence of larger pores between crystals from needle-like structures compared to plate-like structures that enhances permeability, and the selective exposure of specific crystal faces.^122^ Furthermore, the affinity for certain proteins was dependent on the crystal morphology; for instance, a preferential adsorption of proteins involved in the clotting cascade during inflammation and bone remodelling was observed for higher aspect ratio nanostructures compared to plate-like ones, which might favour tissue regeneration. It has also been shown that protein interaction and modulation is highly triggered by the dimension, morphology, and surface charge of HA.^123^ Due to the hydrated layer formed and surrounding the HA surface upon contacting fluids, the ionic exchange which is exacerbated with higher surface area in high aspect ratio structures can influence protein conformation as well.^124^ Another important feature in high aspect ratio nanostructured HA, with regards to protein interaction, is the preferential exposure of ions in the crystal oriented lattice. For instance, high aspect ratio *c*-axis oriented HA preferentially exposes the crystallographic plane rich in Ca^2+^ ions. In that sense, electron charge density quantifications have reported a positive charged surface in (0 0 1) plane, whereas (1 0 0) plane resulted in a negative charged surface.^125^ This has a strong influence in the type of proteins adsorbed whose concentrations can greatly impact the recruitment of cells. Indeed, growth factors recruitment at the interface of materials upon contact with fluids has been shown to trigger cell engagement and favour tissue formation. More recently, in hands with the development of 3D printing technologies enabling the control of scaffold geometry, pore sizes and porosity at the macroscale level, further combining 3D printing with high T and P synthesis, such as hydrothermal or solvothermal methods, has helped to further tune the same features at the micro and nanoscale level, although still with a certain stochastic nanostructure formation. For instance, high aspect ratio HA obtained *via* hydrothermal method has shown to produce whiskers with enhanced protein adsorption profiles, improved wettability, compressive strength and stiffness compared to low aspect ratio structures.^73^ Similarly, hydrothermally processed 3D printed scaffolds yielding high aspect ratio nanostructured HA exhibited a higher protein adsorption and penetration throughout the scaffold compared to lower aspect ratio HA (Fig. 8).^69^

#### 5.2.2. Cell and tissue interactions with high aspect ratio hydroxyapatite

From a physical perspective, the high aspect ratio structures can have a strong effect on cellular attachment. As eukaryotic cells come in contact with the structural features in nanostructured materials, their adhesion and motility can be highly affected.^114^ This is relevant when the dimensions of the nanostructures have length scales similar to cellular components, and it has been demonstrated that the diameter, spacing and uniformity, composition and secondary structure of nanostructured high aspect ratio surfaces interfere in cellular interactions.^114^

Importantly, these topographical features regulate initial cell attachment. As a general rule, submicron roughness is known to increase cell attachment compared to smooth surfaces, although the threshold for up or downregulation of cell attachment can greatly vary between materials due to other properties such as their chemical composition or their mechanical properties such as stiffness.^126,127^ On the other hand, less bioactive materials than HA such as polystyrene, silicon or titanium with high-aspect-ratio nanopatterns have shown a reduction in cell spreading, although improving adhesion. This reduction in cell spreading later impaired cell proliferation and maturation. This phenomenon was concluded to be due to a disorganization of the cell cytoskeleton and overproduction of filopodia, which reduced the integrin clustering, focal adhesion points in eukaryotic cells, necessary for cell signalling and transduction for later new tissue formation.^128,129^ However, another combinatorial study found that increasing the aspect ratio while maintaining the spacing between nanostructures led to increased cell polarization and alignment.^130^ These findings highlight the importance of finding a balance between high aspect ratio stimulation *versus* disincentive effects by accurately controlling the design of such nanostructures in terms of spacing, length, diameter, and height. It is important to note that, in addition to the direct topographical effect, there may be a mechanical effect associated with the variation in the flexibility of the high aspect ratio nanostructures. High aspect ratio nanostructures can have a high flexibility, resulting in ‘overall softer’ surfaces, which can be key to positively control cell fate and progression.^130^

**Fig. 9:**
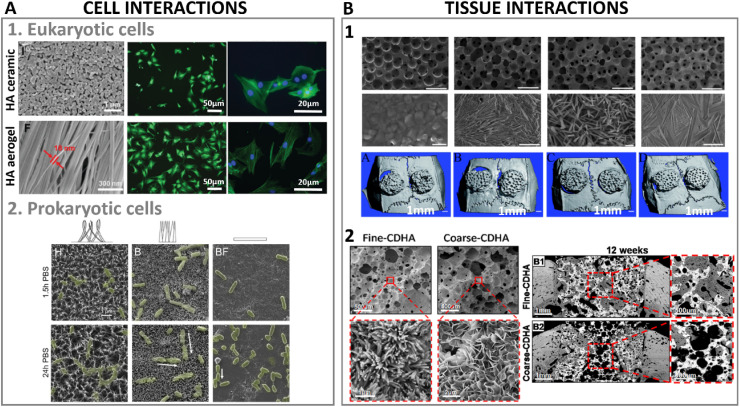
(A1) High aspect ratio HA nanostructures' interactions at cellular level with eukaryotic cells (adapted with permission from ref. 140, copyright 2021 Royal Society of Chemistry), and (A2) culture of HA nanostructured surfaces with prokaryotic cells exhibiting mechano-bactericidal properties (adapted with permission from ref. 141, copyright 2022 Elsevier). (B1) Bone regenerative ability of high aspect ratio HA at a tissue level interaction in a calvarian rat defect after 8 weeks of implantation (adapted with permission from ref. 135, copyright 2013 Royal Society of Chemistry), and (B2) nanostructured HA implanted in a femoral section depicting bone regeneration in a canine model after 12 weeks (adapted with permission from ref. 133, copyright 2019 American Chemical Society).

HA biomaterials for regenerative medicine applications are often obtained in the form of scaffolds, porous structures that support tissue growth. As described in Section 4.2 this can be done by 3D-printing technologies, and a post-processing step can be introduced to obtain high aspect ratio nanostructured HA. In correlation with the higher protein adsorption profiles of nanowires or needle-like HA, increased cell proliferative rates and tissue regeneration have been found for these type of high aspect ratio nanostructures.^69,73^ Similarly, larger aspect ratio nanorods were found to promote bone tissue formation when combined with sodium alginate compared to same composition nanorods with lower aspect ratio.^131^ In recent years, our group has developed high aspect ratio nanostructures through biomimetic precipitation with enhanced osteoinductive properties, capable of bridging critical size defects in large animal orthotopic and ectopic bone models.^132,133^ High aspect ratio needle-like nanostructured HA exhibited enhanced bone formation compared to low aspect ratio plate-like crystals.^133^ The smaller crystals in needle-like structures contributed to larger specific surface area, increasing surface reactivity and improving cell-mediated degradation of the scaffolds and promoting new bone formation (Fig. 9B2). Similar scaffolds with higher aspect ratio crystals were obtained *via* hydrothermal setting, and compared to a biomimetic setting. Larger specific surface area was obtained *via* hydrothermal setting, providing higher aspect ratio HA crystals which outperformed the new bone formation compared to nano-sized plate like crystals using identical 3D printed pore networks.^69^

Nanofibrous ceramics obtained by solvothermal synthesis have also been tested in terms of protein and cell interactions. An elastic sponge combining tricalcium phosphate fibres with HA nanowires reported almost 40% higher protein adsorption using haemoglobin as model protein when HA nanowires were present.^62^ All these studies have commonly agreed at pointing at the higher recruitment of growth factors and cells due to the higher aspect ratio structure as key for new tissue formation. Other approaches have utilised ice templating combined with high aspect ratio HA fibers to obtain controlled macroporous structures with aligned nanostructures with high aspect ratio features. A combination of HA nanowires with beta-tricalcium phosphate fibers resulted in a fully inorganic flexible sponge capable of enhancing protein adsorption, while improving cell penetration throughout the scaffold volume, thus increasing cell proliferation and differentiation.^62^ Similar consolidation methodology, in this case loading magnetic iron oxide nanoparticles onto HA high aspect ratio nanowires, resulted in an on-demand motion purely ceramic scaffold. The magnetic ceramic scaffold had tunable stiffness depending on the HA nanowire content, 50% deformation and high elasticity. Through the control of the magnetic particle distribution in the nanowire network, and a gradient magnetic particle load achieved *via* freeze drying, the scaffolds were endowed with on-demand motion upon magnetic stimulation, which was further explored as mechanism for accelerating drug release upon drug loading.^134^ The use of sacrificial templates to produce macroporous scaffolds has also been employed with subsequent calcination and later combining hydrothermal treatment to produce different aspect ratio nanostructures.^135^ The combination of micro and nanohybrid topography derived from high aspect ratio needle/nanowires enhanced cell differentiation into the osteogenic lineage, and promoted bone regeneration significantly compared to the absence of such high aspect ratio nanofeatures (Fig. 9B1).

The effect of the aspect ratio of HA on the inflammatory response has also been a field of interest in regenerative medicine due to the signaling role of immune cells to trigger other cells' responses, and promote tissue healing. Higher aspect ratio needle-like HA was shown to induce less release of reactive oxygen species (ROS) by immune cells compared to plate-like HA crystals; the released ROS can be associated to cell damage, promoting inflammation and causing cell death.^136,137^ Similar substrates with high and low specific surface area, consisting of needle or plate-like HA crystals were studied with both immune and bone cells, and the osteoimmunomodulatory properties of the substrates were reported.^138^ High aspect ratio nanostructure HA (needle-like) was found to stimulate the production of pro-inflammatory cytokines, which later triggered a more favorable osteogenic response. Nevertheless, in some cases, high-aspect-ratio nanostructured HA has also been reported to cause cell damage. A study using immune cells depicted a reduced viability when high aspect ratio HA was used in contrast to smooth HA plasma sprayed surfaces with a less nanostructured features. However, the higher the aspect ratio of HA structure, the less cell viability reduction when comparing lengths of 170 nm *vs.* 110 nm length.^139^

An additional advantage of obtaining high aspect ratio HA for bone regenerative strategies is the possibility to overcome a recurring limitation, *i.e.* the brittleness and fragile behavior of bioceramic scaffolds. The use of HA nanowires comprising aspect ratios even as high as 1 : 10 000 has shown enhanced tensile strength and flexibility.^50^ Additionally, the combination with more flexible materials such as collagen, alginate or chitosan has further proven the feasibility of obtaining shape-recovery scaffolds with almost a fully elastic behaviour.^62,111,140^ These elastic mechanical properties were obtained in an aerogel of flexible HA nanowires, that was shown to promote bone formation. The alignment of the fibers/nanowires in combination with the macropores obtained by ice templating was crucial to achieve higher cell adhesion, proliferation and differentiation, and neovascularization, which turned into enhanced new bone formation (Fig. 9A1).^140^

### 5.3. Interaction with prokaryotic cells: mechano-bactericidal surfaces

Similar to eukaryotic cells, prokaryotic cells interact with materials surfaces, although in the case of prokaryotic cells, the desired outcome of their interactions with materials' surface might be the opposite. Since the early discovery of the bactericidal effect in insects' wings,^142^ the use of nanostructured surfaces has gained interest as bactericidal approaches for antibiotic free materials, especially with the increasing concerns regarding bacterial antibiotic resistance.^143^ Usually, bacterial adhesion occurs in two steps: first, a physicochemical interaction to initial attraction takes place, then a firmer adhesion by selective bridging of bacteria polymeric structures (adhesins) to materials' surface occurs. The first step of bacteria interaction with materials' surface represents a niche of action for bactericidal materials.^144^

Although the mechano-bactericidal mechanisms of high aspect ratio nanostructures remain under debate between damaging bacteria during settlement or by impairing their mobility, it is certain that high aspect ratio nanostructured surfaces have the ability of inducing mechanical stresses in bacteria membrane, either during adhesion or division, owing to the similar length scale of the nanostructures to those of bacteria cellular components. A recent review by Modaresifar *et al.* summarised the most used patterns and dimensions with bactericidal effects.^145^ They concluded that aspect ratios comprised between 0.3 and 100, with spacing between the nanostructures limited below 500 nm were effective in killing bacteria. Importantly, depending on the application and desired outcome, certain nanostructured surfaces are intended to have a dual claim of offering bactericidal effects while still warranting eukaryotic cell survival.^146^ Noteworthy, bacterial strains can differ in their cell membrane arrangement. For instance, Gram-positive bacteria membrane consists of a thick peptidoglycan layer (for instance, *S. aureus* cell wall was found to be 10 nm), whereas Gram-negative bacteria membrane possesses a thinner peptidoglycan layer and a lipopolysaccharide layer (cell wall thickness of 6 nm and 3 nm for *P. aureginosa* and *E. coli*, respectively).^147,148^ These particularities in cell membrane make are translated in higher forces required to disrupt *S. aureus* for instance due to the thicker cell wall,^149^ which have helped establishing turgor pressure thresholds for effectively killing different bacterial strains.^150^ Hence, the mechano-bactericidal effects of high aspect ratio nanostructures need to be applicable preferably for indistinctive bacteria types; however, it has only been recently that biomimetic and biocompatible HA based materials have been explored for their mechano-bactericidal application. A recent study using HA synthesised *via* hydrothermal and biomimetic precipitation differing in their interspacing and height of high aspect ratio needles reported 70% bacterial death after 24 h of contact with these surfaces. A slightly higher bactericidal effects were observed when the needle high aspect ratio structures had broader interpillar and height distributions (Fig. 9A2).^141^

High aspect ratio HA offers an example of highly hydrophilic surface, where bacterial attachment might likely occur, although wettability of high aspect ratio HA can be tuned as explained in Section 5.1. A recent study highlighted that although hydrophobic surfaces are highly efficient in killing bacteria, the same outcome can be reached on hydrophilic ones although at expenses of longer incubation times.^151^ Noteworthy, in biological applications where tissue regeneration is sought, hydrophilicity is paramount for eukaryotic cell survival.

Strategies to chemically modify hydrophilic HA with antibacterial ions such as Ag or Zn,^152^ in combination with high aspect ratio conformation have shown enhanced antibacterial properties. As described in previous sections, applications of synthetic paper and membranes have been combined with antibacterial ions to treat polluted water, demonstrating high efficiency in reducing bacterial growth when high aspect ratio HA, silver, zinc, and even antibiotics (ciprofloxacin) are used.^92,93^ Another antibiotic free approach based on nanostructured HA coating on titanium meshes reported a decrease in bacterial adhesion and growth, and further studied the inflammatory response of the proposed surfaces. Macrophages, immune cells, depicted a reduced viability when high aspect ratio HA was used in contrast to HA plasma sprayed surfaces with a less nanostructured features. However, longer HA structures, with higher aspect ratio, were more permissive than shorter counterparts (170 nm *vs.* 110 nm length).^139^ A step further, another study using fluorhydroxyapatite co-doped with Ag and Sr found antibacterial effects up above 95% while warranting cell survival (osteoblast cells) and new tissue formation.^75^ The combination of high aspect ratio nanostructured HA with the ionic release of silver, for bactericidal effects, and strontium, to counterbalance any possible silver toxicity to mammalian cells, was pointed as key is the results.

## 6. Future outlook

High-aspect-ratio nanostructured HA is attractive for a wide range of applications. Throughout this article we have highlighted a number of recent studies that have demonstrated that, by leveraging their unique properties, it has the potential to improve performance and efficiency in different fields, including biomedical implants, drug delivery systems, catalysis, energy storage, and environmental. However, many challenges remain before the full potential of these materials can be realised.

The synthesis of high-aspect-ratio HA requires a precise control of crystal morphogenesis, which in turn demands a deep understanding of the fundamental mechanisms of crystal growth identifying appropriate additives, and achieving reproducibility. Despite significant progress, to a large extent thanks to studies in the field of biomineralisation, developing predictive models that can accurately predict crystal morphology remains a significant challenge. Such models require taking into account complex and dynamic interactions between the crystal, the solvent, and any additives, in addition to a wide range of factors, including temperature, pressure, pH, and reaction time among others. Moreover, identifying appropriate additives that can be used to control crystal morphology by altering nucleation and growth rates can be challenging. Finally, developing methods for maintaining uniformity and reproducibility in crystal morphology is essential for producing materials with consistent properties. Finally, scalability is another issue, since the synthesis of high aspect ratio HA is typically performed in small batches, making it challenging to scale up production for industrial applications.

Regarding the different applications, there is still a broad space for future research for tailoring the textural and surface properties of high-aspect-ratio HA, such as size, shape, and surface chemistry, to meet specific requirements in fields as varied as drug delivery, tissue engineering, or catalysis, with a view of making the most of the crystal morphology for each specific application. For instance, high-aspect-ratio HA has been shown to interact with biological systems, such as cells and proteins, in unique ways. However, although some trends have been revealed, more research is needed to identify the most appropriate morphologies, in terms of *e.g.* aspect ratio, size or surface chemistry, to trigger specific biological outcomes, such as cell differentiation or inflammatory responses. The prospects are also very promising if we look at the mechanical properties of these materials. The production of flexible ceramics opens up new perspectives, and their possible combination with other materials, especially polymers, to produce hybrid materials, multiplies the possibilities.

Finally, it would be inaccurate to think of a future perspective without considering the enormous potential offered by artificial intelligence (AI) tools. The enormous advances in AI are expected to play a prominent role in the coming years in the field of inorganic materials synthesis, and specifically in the control of crystal morphology, as it is happening in so many other fields.^153^ Most research to date in the field of inorganic materials synthesis has been carried out on a trial-and-error basis, and although a large amount of data has been generated, its heterogeneity and the disparity of experimental conditions often make it difficult to extract information in a consistent manner. With AI, researchers can use algorithms to analyse large amounts of data and identify patterns that otherwise would be difficult for humans to discern. For example, a recent study demonstrated that natural language processing techniques can be used to automatically extract synthesis information from zeolite journal articles and analyse the complex relationships between the synthesis parameters and resulting topology.^154^ Moreover, machine learning strategies allow predicting synthesis pathways for new zeolite structures. A similar approach has been extensively applied to predict the morphology of metal–organic frameworks crystals based on synthesis conditions.^155,156^ Although so far this strategy has not been applied for the control of crystal morphology of nanostructured HA, these studies illustrate the enormous potential of machine learning strategies for the development of predictive models for materials synthesis, capable of embracing the complexity behind the synthesis process, and their relationship to the morphology of the resulting materials, which is precisely the key in the development of tailored HAR HA.

## Author contributions

Maria-Pau Ginebra outlined the first draft and contribute to writing Sections 1, 2, 3, and 6. Montserrat Espanol contribute to writing Section 4. Anna Diez-Escudero contribute to writing Sections 4 and 5. Maria-Pau Ginebra and Anna Diez-Escudero designed and produced the figures. All authors reviewed the manuscript and provided feedback for the final version.

## Conflicts of interest

There are no conflicts to declare.

## Supplementary Material
